# Designer receptor technology for the treatment of epilepsy

**DOI:** 10.1016/j.ebiom.2019.04.059

**Published:** 2019-05-09

**Authors:** Andreas Lieb, Mikail Weston, Dimitri M. Kullmann

**Affiliations:** Department of Clinical and Experimental Epilepsy, UCL Queen Square Institute of Neurology, University College London, UK

**Keywords:** Designer receptor, Chemogenetics, DREADD, hM4D(Gi), GluCl, PSAM

## Abstract

Epilepsy remains refractory to medical treatment in ~30% of patients despite decades of new drug development. Neurosurgery to remove or disconnect the seizure focus is often curative but frequently contraindicated by risks of irreversible impairment to brain function. Novel therapies are therefore required that better balance seizure suppression against the risks of side effects. Among experimental gene therapies, chemogenetics has the major advantage that the action on the epileptogenic zone can be modulated on demand. Two broad approaches are to use a designer G-protein-coupled receptor or a modified ligand gated ion channel, targeted to specific neurons in the epileptogenic zone using viral vectors and cell-type selective promoters. The receptor can be activated on demand by either an exogenous compound or by pathological levels of extracellular glutamate that occur in epileptogenic tissue. We review the principal designer receptor technologies and their modes of action. We compare the drawbacks and benefits of each designer receptor with particular focus on the drug activators and the potential for clinical translation in epilepsy.

## Highlights

• Inhibitory designer receptors (DRs) allow on-demand suppression of seizures upon activation by exogenous drugs or endogenous neurotransmitters.
• DRs include modified G-protein coupled receptors, chimaeric ligand-gated ion channels, and mutated non-mammalian channels.
• Identification of drug activators of DRs that are already approved for use in humans significantly accelerates clinical translation.

## 1 Introduction

Despite the introduction of over fifteen new antiepileptic drugs in the last twenty years, the proportion of people with epilepsy whose disease is refractory to treatment remains largely unchanged: approximately 30% of patients continue to experience seizures even with optimal medical treatment [1,2]. These patients suffer from a devastating impact on their quality of life, and are exposed to a substantial risk of sudden unexpected death (SUDEP), estimated at 0.14% per year and twenty-three-fold higher in comparison to the unaffected population [3]. Refractory epilepsy is in the majority of cases associated with focal seizure onset, which may generalize. Surgical resection of the epileptic zone is currently the only treatment option that offers a reasonable prospect of seizure freedom but is contraindicated for a substantial proportion of patients because of unacceptable risk of irreversible and severe consequences from removal of brain tissue for memory, language, motor or sensory function. Newer less invasive strategies such as laser-mediated treatment of the epileptogenic zone are also destructive and irreversible. Alternative treatment strategies are therefore urgently required.

Gene therapy, achieved via intraparenchymal injection of a viral vector, is arguably the most promising treatment strategy to address this unmet need. It relies on the expression of various proteins to prevent seizure initiation or propagation in the targeted brain region. Because the rest of the brain is unaffected, the risk of side effects is minimized. Furthermore, by biasing expression of transgenes to specific subtypes of neurons (typically excitatory principal cells), it is possible to exploit knowledge of the normal mechanisms underlying the excitation-inhibition balance, and how this is altered by pro- or anti-epileptic drugs, to design rational treatments.

Several gene therapy strategies have been validated in preclinical models, including the overexpression of endogenous neuropeptides [[4], [5], [6], [7]] and potassium channels [8]. Some of them are amenable to clinical translation [9]. Nevertheless, a potential limitation of these approaches is that gene transfer to neurons is irreversible, and it may be difficult to identify the optimal dosage to achieve a therapeutic effect without compromising normal brain function. Indeed, dosage of viral vectors consists both of viral copy number per infected neuron, and the number of cells infected, and needs to be tailored precisely to ensure that the epileptogenic zone is effectively treated with minimal spread to neighbouring or overlapping regions of eloquent cortex. When the seizure focus is diffuse, or overlaps extensively with regions controlling language, memory, motor or sensory function, the therapeutic window for gene therapy may be very narrow. These concerns underpin the need to identify gene therapy strategies whose effect on neuron or circuit function can be adjusted or even switched on and off on demand.

The ability to switch an experimental anti-epileptic gene therapy on and off was first reported with optogenetics [8,10,11]. This approach has high temporal specificity: the anti-seizure effect can be switched on within less than a second. It can also be used in closed loop, where light delivery is triggered as soon as a seizure, or an electrographic signature of an impending seizure, is detected. Several approaches have been proposed, including optogenetic hyperpolarization of principal neurons or depolarization of inhibitory interneurons, although the latter approach can sometimes have paradoxical effects [12]. Optogenetics however presents major translational obstacles, because of the need to express non-mammalian proteins in the brain and to implant devices for illumination of the opsins. These concerns justify interest in chemogenetics as an alternative strategy for on-demand anti-epileptic gene therapy.

**Fig. 1:**
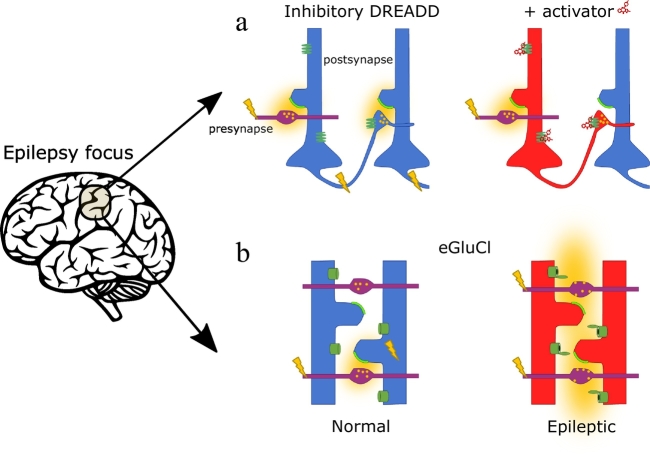
Overview of DRs used to treat epilepsy. Illustration of the mechanisms of chemogenetic anti-epileptic strategies. **a**: Neurons transduced with an inhibitory DREADD (such as hM4D(Gi)) are less excitable and fail to release neurotransmitter in the presence of an activator (such as CNO or Olanzapine). The net result is to prevent seizure propagation. **b**: The autoregulatory receptor eGluCl opens a chloride conductance in response to pathological extracellular glutamate elevation (yellow clouds) as occurs with intense afferent activity, inhibiting neurons. In both schematics, afferent axons are indicated in purple, blue neurons illustrate cells that are unaffected by the chemogenetic treatment because the receptors are not bound by ligand (exogenous in **a**, endogenous glutamate in **b**), and red neurons illustrate the effect of the chemogenetic inhibition. Green helix: hM4D(Gi)-DREADD, Green cylinder: eGluCl. (For interpretation of the references to colour in this figure legend, the reader is referred to the web version of this article.)

Chemogenetics can be broadly defined as the use of engineered receptors to confer a pharmacological sensitivity to cells that they do not normally exhibit. The most widely used chemogenetic tools include Designer Receptors Exclusively activated by Designer Drugs (DREADDs) derived from G-protein-coupled receptors (GPCRs) [13], and synthetic ligand-gated ion channels (LGICs) [[14], [15], [16], [17], [18]]. These receptors are activated by exogenous compounds, and when expressed in neurons can either inhibit or excite them (Fig. 1). The most promising chemogenetic strategies to treat epilepsy are described in the following sections, together with their potential for clinical translation, summarized in Table 1.

**Table 1:** Overview, advantages and disadvantages of chemogenetic silencing tools for use in epilepsy.

Tool	Ligand	Pros	Cons
DREADDs			
hM4D(Gi)		• Mutated human receptor: immunogenicity unlikely	• Risks of desensitization and basal activity not assessed in humans
	CNO	• Metabolite of a clinically approved drug	• Short half-life • Not clinically approved • Proportion metabolised to clozapine in humans, non-human primates and rodents
	Clozapine	• Clinically approved	• Risk of precipitating seizures • Unpredictable risk of bone marrow suppression
	Perlapine	• Previously approved in Japan	• Not currently clinically approved
	Compound-21	• Potent activator	• Not clinically approved • Affinity to Histamine H1 receptor
	Olanzapine	• Potent activator • Clinically approved	• Main side effects: weight gain and drowsiness
KORD	Salvinorin B		• Activates native opioid receptor at relatively low concentrations • Not clinically approved • Side effect profile unknown
RASSL	Spiralodine		• Ligand activates native receptors • Not clinically approved
Alstr	Allastatin		• Not clinically approved • Ligand does not cross the blood brain barrier

LGIC-DRs			
eGluCl	Glutamate	• Autoregulation • No need for additional drugs • Add-on therapy possible, to allow scaling of the therapeutic effect	• Potential immunogenicity, although not reported in non-human primates • Therapeutic window depends on pathological extracellular glutamate being much higher than during normal glutamatergic signalling
eGluCl GluClv2.0 GlyR-DR	IVM	• Clinically approved drug with well-known side effect profile • Blood-brain barrier breakdown during seizures may increase local IVM concentration, enabling autoregulation	• Potential immunogenicity, although not reported in non-human primates • IVM not clinically approved for treatment of epilepsy and sub-optimal pharmacokinetics • Risk of heteromerization with native receptors
PSAM/PSEM	PSEM^89S^		• Ec_50_ in micromolar range • PSEM^89S^ effect on normal brain function not reported • Short half life • Requires high doses in vivo • Risk of heteromerization with native receptors
	PSEM^308^		• Unknown side effect profile • Risk of heteromerization with native receptors
PSAM4	Varenicline	• EMA/FDA licensed drug with known pharmacology	• Not clinically approved for treatment of epilepsy • Risk of nausea, abnormal dreams, insomnia • Risk of heteromerization with native receptors

## 2 GPCR-based DREADDs

The fundamental principle underlying DREADDs is that the engineered receptor has been mutated to render it insensitive to the normal endogenous ligand (designer receptor), but sensitive to one or more exogenous compounds that otherwise have no effects on the tissue (designer drugs). GPCRs mutated to alter the selectivity of the ligand-sensing domain were created almost three decades ago [19,20]. However initial versions, termed Receptors Activated Solely by Synthetic Ligands (RASSLs), were limited by retained affinity for the native ligand, and/or constitutive activity with high levels of receptor expression [21]. The Drosophila allastatin GPCR was an improvement but its ligand is unlikely to cross the blood brain barrier (BBB) [22].

A breakthrough came in 2007 when Armbruster et al. used directed molecular evolution of the human M3 muscarinic receptor (hM3) to render it insensitive to its endogenous ligand acetylcholine, while imparting potent sensitivity to the ligand Clozapine-N-Oxide (CNO), an inert metabolite of the atypical antipsychotic drug clozapine [13]. On exposure to CNO, neurons transduced with this mutated Gq-coupled-DREADD (hM3Dq) exhibit intracellular calcium release and depolarisation, corresponding to an increase in excitability. Only two amino acids in the ligand-binding domain were altered from the parent hM3 receptor, and this region is highly conserved across muscarinic receptors, allowing the same mutations to generate hM4D(Gi), based on the related human M4 inhibitory Gi-coupled DREADD. Neurons transduced with hM4D(Gi) and exposed to CNO exhibit hyperpolarization mediated by opening of G-protein sensitive inwardly rectifying potassium channels (GIRKs) [13]. HM4D(Gi) activation also leads to a decrease in neurotransmitter release from presynaptic terminals [23,24].

Further work led to other DREADDs: 1) a chimeric M3-derived receptor with intracellular loops from the turkey β1 adrenergic receptor, activation of which leads to a Gs-mediated increase in cAMP [25]; 2) M3Dq-R165L which initiates β-arrestin signalling [26]; 3) hM4D-neurexin, a presynaptic inhibitory DREADD targeted to axons [23]; and 4) a κ-opioid derived DREADD (KORD) coupled to the Gi cascade and activated by the otherwise pharmacologically inert compound salvinorin B [27].

These tools have had an enormous impact on circuit neuroscience [21,28]. To date, most studies have used hM3Dq or hM4D(Gi) for on-demand neuronal excitation or inhibition respectively. KORD can be multiplexed with hM3Dq/hM4D(Gi) to allow excitation/inhibition in specified neuronal subtypes of different brain regions, or even within loci.

**Fig. 2:**
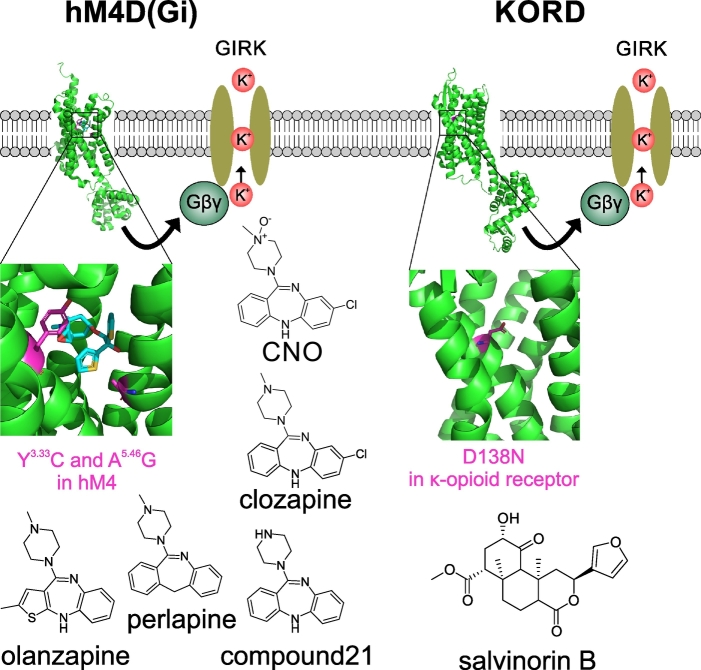
GPCR-based chemogenetic silencing tools. Activation of GPCR-based chemogenetic silencing tools (hM4D(Gi) and KORD) leads to dissociation of Gβγ G-protein subunits, which then lead to opening of GIRKs. Gαi dissociation also leads to inhibition of adenylyl cyclase and a reduction of neurotransmitter release. **HM4D(Gi):** Mutations introduced into the human M4 muscarinic receptor (hM4) are highlighted (magenta) in the crystal structure of hM4 in complex with Tiotropium, an antimuscarinic drug (cyan) (PDB entry 5dsg [75],). The structures of known potent agonists of hM4D(Gi) are shown below. **KORD:** Mutations introduced in the κ-opioid receptor crystal structure (PDB entry 4djh [76],) are highlighted (magenta), together with the structure of the selective agonist salvinorin B. (For interpretation of the references to colour in this figure legend, the reader is referred to the web version of this article.)

For basic neuroscience studies an important determinant of the utility of different DREADDs is how selectively individual compounds are able to activate them, and whether the ligands can be delivered systemically or instead require local application. For instance, salvinorin B activates the native κ-opioid receptor at relatively low concentrations (100 nM) which is only about eight-fold higher than the EC_50_ of overexpressed KORD [21,27]. The most extensively used inhibitory DREADDs and their activators are shown in Fig. 2.

### 2.1 Inhibitory DREADD treatment of epilepsy

DREADDs offer the potential of clinical translation [21,29], in particular to treat epilepsy because of decades of work supporting the fundamental principle that manipulating the excitation-inhibition balance either triggers or suppresses seizures. Kätzel et al. first reported that seizures, both in an acute rodent chemoconvulsant model and in a model of chronic epilepsy, could be suppressed by activating hM4D(Gi) expressed in excitatory neurons, using intraperitoneal injection of CNO as the activating ligand [24]. Several subsequent studies have used hM4D(Gi) as an experimental tool to examine the circuits underpinning seizures, both in vitro [30] and using a kindling model of rodent epilepsy [31,32], helping to identify the critical nodes of epileptogenic networks. These studies have used viral vectors to drive expression of hM4D(Gi) in excitatory neurons to suppress seizures, consistent with the simple assumption that inhibiting excitatory cells should be anti-epileptic. The prediction that silencing inhibitory hippocampal interneurons with hM4D(Gi) should be pro-epileptic has also been supported experimentally [33].

Accumulating evidence supports the use of DREADDs as an effective tool to suppress excitatory hippocampal neurons using hM4D(Gi) in other rodent models of chronic epilepsy. In both the mouse intrahippocampal kainic acid model [34] and the mouse intraperitoneal pilocarpine model [35] there was a significant reduction of seizure frequency with daily administration of CNO. Conversely, the pro-excitatory DREADD hM3Dq, expressed in inhibitory parvalbumin-positive interneurons, may also be effective. Activation of interneurons using this strategy suppressed epileptiform synchronization [36], and it has recently been shown that this both extends the latency to seizures in a kindling model and significantly reduces seizures in the mouse intrahippocampal kainic acid model [37]. Interestingly, the seizure reduction was equivalent to that induced by hM4D(Gi)-mediated inhibition of excitatory cells. Additionally, irrespective of which DREADD and cell type combination was used, epileptic animals performed poorly on memory assays with or without CNO, likely a reflection of the cognitive effects of hippocampal epilepsy and not DREADD efficacy [37].

Thus, in multiple rodent epilepsy models, DREADD technology has been shown to be effective at reducing seizure frequency and/or propagation when the engineered receptors are appropriately targeted to seizure foci or nodes in the network. As the anti-seizure effect requires the presence of an exogenous activator, it is reversible and consequently potentially represents a major advantage for clinical translation to treat focal epilepsy. By allowing the degree of inhibition to be fine-tuned, the risk of permanent cognitive impairment engendered by resective surgery is removed. Indeed, even if an effective anti-seizure effect cannot be dissociated from an effect on normal brain function, DREADD-based treatment could still, in principle, be useful: some patients have such severe epilepsy that they experience episodes of status epilepticus requiring intravenous sedation, endotracheal intubation and artificial ventilation on the intensive care unit, with appreciable mortality. If pre-treated to express a DREADD in the appropriate cell type and brain region (most simply, an inhibitory DREADD in the excitatory neurons of the epileptogenic zone), the selective agonist could be administered in the emergency room to terminate status epilepticus. A temporary effect on language, memory, motor or sensory functions could be an acceptable side effect of treatment to give time to optimize other anti-epileptic medication or consider further interventions.

There are, nevertheless, special considerations to take into account when considering the use of DREADDs in humans. Many GPCRs desensitize on repeated activation, and relatively little is known about the ability of DREADDs to suppress seizures in the long term. In principle, if they lose their effect with chronic ligand delivery, this may limit their suitability as a maintenance treatment for epilepsy as opposed to on-demand short-term treatment. There are, however, reasons to believe that this is unlikely to be a serious limitation. First, another Gi-coupled GPCR, the GABA_B_ receptor, mediates the action of the anti-spacticity drug baclofen, which is widely used in neurological practice, in many cases for years or decades with no evidence of tachyphylaxis. Indeed, repeated daily dosing of CNO sufficient to substantially reduce seizures has not demonstrated marked tachyphylaxis in a rodent model [34], albeit followed only over a few days. Second, GPCRs, unlike ion-channel based chemogenetic strategies, use a secondary messenger cascade to amplify the intracellular signal. Thus if sufficient chemogenetic receptors and secondary messengers are present, full activation of the cascade is still possible, even if a proportion are desensitized, a phenomenon known as receptor reserve [21].

For clinical translation of muscarinic DREADDs in epilepsy, the choice of activator is critical. CNO is not a drug that has been approved for use in humans by the Food and Drug Administration (FDA) or European Medicines Agency (EMA), even though, as a metabolite of clozapine, it is present in patients treated with clozapine for psychosis. Recent research has moreover shown that CNO does not pass the rodent blood brain barrier (BBB), and instead probably acts on DREADDs expressed in the CNS by being back-converted to clozapine, which enters the brain [38]. This likely also occurs in non-human primates [21]. Could clozapine itself be used as a DREADD activator in humans? Clozapine has a complex pharmacology, acting on a range of dopaminergic, muscarinic, serotonergic and histaminergic receptors at low concentrations. It would therefore not be a selective DREADD agonist in this setting, but if the off-target effects mediated by other receptors were tolerable it could in principle lower the barrier to clinical translation. In reality, the side effect profile of clozapine is highly unfavourable for use in epilepsy, because it has a substantial risk of bone marrow suppression and can lower seizure threshold in some people [39]. Although it may be possible to use very low doses of clozapine to minimize these side effects, identification of alternative agonists would facilitate clinical translation of muscarinic DREADD technology. Other brain-penetrating agonists, “compound 21” and perlapine, have been described [40,41]. While both activate hM4D(Gi) at low nanomolar concentrations and penetrate the BBB, neither is currently approved for use in humans by the FDA or EMA. Perlapine has actually been used in the clinic as a sedating antihistamine in Japan and a few other countries but was withdrawn from the market without undergoing FDA or EMA scrutiny. In addition, it has recently been shown on radioligand assays that both CNO and compound 21 also bind to other receptors at low nanomolar concentrations [42]. Identifying an alternative agonist that is already FDA/EMA-approved would substantially accelerate the path to clinical trials testing the safety and efficacy of muscarinic DREADD therapy of CNS disorders.

We recently showed that olanzapine, another FDA/EMA-approved antipsychotic drug with a better safety profile than clozapine, is a potent agonist of hM4D(Gi) [43]. By measuring the ability to open G-protein coupled potassium channels in vitro, we estimated an EC_50_ at hM4D(Gi) around 5 nM, 10-fold lower than for clozapine (EC_50_ ~50 nM). When tested in vivo at 0.1 mg/kg it reduced the latency to falling off a RotaRod in mice with widespread hM4D(Gi) brain transduction, whilst clozapine at the same dose was ineffective [43]. The most common side effects of olanzapine reported in patients treated for psychosis are weight gain and drowsiness, with substantially reduced risk of precipitating seizures compared to clozapine [39]. A clinical trial of hM4D(Gi) activated by olanzapine to treat focal epilepsy would therefore seem the most promising strategy for clinical translation of DREADD technology.

## 3 Ligand-gated ion channels

LGICs are drug targets for many CNS diseases including epilepsy [44]. LGICs can broadly be divided into two major classes depending on whether they permeate cations or anions. Cation-permeable LGICs promote depolarization and neuronal excitation. Excitatory LGICs in the mammalian CNS include: 1) cation-conducting nicotinic acetylcholine receptors and 5-HT_3_ serotonin receptors, which are pentameric; 2) the structurally unrelated tetrameric ionotropic glutamate receptors (AMPA, kainate and NMDA receptors); and 3) trimeric P2X purinergic receptors. Anion-permeable LGICs include pentameric GABA_A_, GABA_C_ and glycine receptors (GlyRs), which have a similar structure to nicotinic receptors [45]. They inhibit neurons both by shunting excitatory currents and, depending on the trans-membrane chloride gradient, making the membrane potential more negative (hyperpolarization).

GABA_A_ receptors are established targets for benzodiazepines and barbiturates, which are widely prescribed antiepileptic drugs. They also mediate at least part of the anti-seizure effects of vigabatrin and tiagabine, which elevate ambient GABA levels. Overexpressing GABA_A_ receptors in excitatory neurons of the epileptogenic zone would therefore seem an obvious strategy to increase the potency of these agents. However, GABA_A_ receptors are heteromultimeric, raising the possibility that overexpression of individual subunits would interfere with normal GABAergic transmission, potentially leading to mislocalization of receptors. Indeed, depending on which subunits are rate-limiting for synaptic or extrasynaptic GABA_A_ receptor expression, this strategy could have unexpected effects on both normal GABAergic signalling and the action of exogenous drugs.

Two strategies have shown promise in developing chemogenetic manipulation of circuit excitability using LGIC-based designer receptors (LGIC-DRs): non-mammalian LGICs that should not co-assemble with receptor subunits present in the mammalian CNS, and chimeric channels.

### 3.1 Glutamate-gated chloride channels for closed loop chemogenetic seizure supression

Many invertebrate species use glutamate-gated chloride channels as inhibitory receptors at their neuromuscular junction. GluCl is distantly related to the mammalian nicotinic family of LGICs. In contrast to mammalian ionotropic glutamate receptors, and in common with GABA_A_, GABA_C_ and glycine receptors, GluCl permeates chloride when activated. GluCl is the target of the anti-helminthic drug ivermectin (IVM). Recognizing the public health implications of this treatment, the 2015 Nobel Prize in Physiology or Medicine was jointly awarded to William C Campbell and Satoshi Omura.

**Fig. 3:**
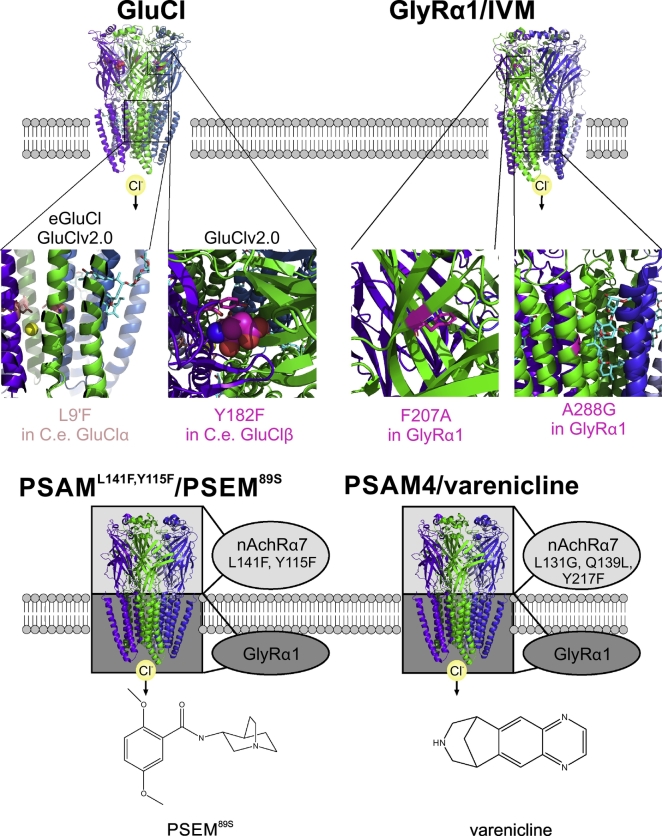
LGIC-based chemogenetic silencing tools. **GluCl**: Mutations that increase the IVM sensitivity (L9’F, orange) and eliminate glutamate activation (Y182F, magenta), are shown in the crystal structure of the *C. elegans* GluCl, in complex with IVM (cyan) and glutamate (magenta spheres) (PDB entry 3rif [77]). **Glyrα1/IVM:** The F207A mutation (magenta), which decreases glycine activation, and A288G (magenta), which increases IVM sensitivity, are shown in the cryo-EM structure of the Glyrα1 in complex with IVM (cyan) (PDB entry 3jaf [78]). **PSAM**^**L141F,Y115Y**^**/PSEM^89S^:** schematic representation of PSAM^L141F,Y115Y^, consisting of the extracellular part of nAchRα7 with mutations that decrease acetylcholine sensitivity and increase the sensitivity to PSEM^89S^, and the trans-membrane and intracellular part of GlyRα1, using the crystal structure of *C. elegans* GluCl as a template (PDB entry 3rif [77]). PSEM^89S^ is shown below. **PSAM4/varenicline:** the mutations increasing varenicline affinity (molecular structure shown below) are shown in a schematic representation using *C. elegans* GluCl as a template (PDB entry 3rif [77]). (For interpretation of the references to colour in this figure legend, the reader is referred to the web version of this article.)

We recently took advantage of the fact that GluCl is normally activated by glutamate to design an autoregulatory chemogenetic treatment of epilepsy that dispenses with the need for an exogenous ligand [15]. Glutamate, the endogenous ligand of GluCl at the invertebrate neuromuscular junction, is also the main excitatory neurotransmitter in the mammalian brain. Normally it is rapidly quenched following exocytosis by abundant perisynaptic glutamate transporters, but during seizures extracellular glutamate concentrations are elevated [46,47]. Extracellular glutamate may also be elevated inside and surrounding gliomas and other lesions that are frequently associated with epilepsy. The principle underlying treatment with GluCl is that an inhibitory chloride conductance opens in the presence of pathologically elevated glutamate, representing an autoregulatory biochemical inhibitory closed loop (Fig. 1). The glutamate sensitivity of GluCl is however in the low millimolar range, whilst extracellular glutamate concentrations are several orders of magnitude lower even in pathological situations. We therefore inserted a point mutation to enhance the glutamate sensitivity (enhanced GluCl or eGluCl) to ~10 μM (Fig. 3). (The same point mutation has been used to increase the IVM sensitivity of GluCl [14]; GluClv2.0, discussed below.) Expression of eGluCl in the rat cortex robustly suppressed seizures in two randomized preclinical animal models of acute seizures and neocortical epilepsy [15].

In principle, GluCl could also be used as part of a conventional chemogenetic inhibition strategy for epilepsy, using IVM or a derivative as the ligand [48,49]. Although IVM opens the native *C Elegans* GluCl at ~140 nM, at much lower concentrations it acts as a positive allosteric modulator (~5 nM) [50]. The same single point mutation that renders eGluCl highly sensitive to glutamate also allows IVM to open it at ~4 nM [14]. A glutamate-insensitive version of GluCl (GluClv2.0) with further codon optimization [51], has been used together with IVM to treat a rodent model of neuropathic pain (Fig. 3) [52], but has not, to our knowledge, been explored as a treatment for epilepsy.

IVM also acts on human GABA_A_ receptors and GlyRs, where it functions as an allosteric modulator at low nM concentrations, and as a full agonist at high nM concentrations [50,53,54]. IVM could therefore be used as an add-on antiepileptic therapy, acting both on eGluCl and on endogenous GABA_A_ receptors and GlyRs [55]. The efficacy and tolerability of this approach would however require further investigation, not least because the pharmacokinetics of IVM are not ideal. It builds up in the brain relatively slowly and has a long half-life, substantially limiting its utility as an on-demand treatment for refractory epilepsy. It is also an mdr-1 substrate, and modulates P2X, G-protein activated inwardly rectifying K^+^ channels, farnesoid X receptors, and α7-nAchR, in addition to GlyRs and GABA_A_ receptors [50,56]. Nevertheless, there is evidence that the blood-brain barrier breaks down temporarily in regions invaded by seizures [57], possibly leading to a greater and more rapid exposure of epileptogenic zones to systemically delivered IVM, and so the pharmacokinetic profile of IVM could actually be an advantage in epilepsy treatment.

A potential obstacle to clinical translation of non-mammalian proteins is that they can trigger an immune response, which has recently been reported for CRISPR-Cas technology [58]. However, GluCl or eGluCl, expressed in the non-human primate or rodent brain respectively, appears to be well tolerated [15,59].

### 3.2 Chemogenetic inhibition with DRs derived from glycine receptors

Another approach to develop a chemogenetic inhibitor used the mammalian glycine receptor as a starting point, mutated to make it sensitive to IVM whilst reducing its sensitivity to glycine [16]. The resulting Gly-DR should represent a lower risk of immunogenicity than GluCl. This technology remains to be tested in epilepsy. A potential concern is that Gly-DR could heteromerize with endogenous glycine receptor subunits. Although synaptic glycinergic transmission appears to be confined to the brainstem, spinal cord and retina, glycine receptors are also present in the forebrain, and so interfering with them could have unexpected consequences [60,61].

### 3.3 Chemogenetic inhibition with chimeric receptors: PSAM/PSEM

A chimeric receptor consisting of the extracellular portions of a mutated α7-nicotinic acetylcholine (nAChRα7) together with the transmembrane and intracellular parts of the GlyR1 glycine receptor subunit has been designed for chemogenetic inhibition using a synthetic ligand. This receptor component of a chemogenetic pair, with amino acid substitutions indicated by superscripts, was denoted the pharmacologically selective actuator module (PSAM^L141F,Y115Y^-GlyR). The selective ligand, on the other hand (with the superscript indicating the order in which molecules were synthesized and tested), was referred to as the pharmacologically selective effector molecule (PSEM^89S^), with a steady-state EC_50_ of 3·4 μM, as compared to an EC_50_ for acetylcholine of 570 μM (Fig. 3) [17]. Although this tool has been exploited in numerous studies of fundamental brain function it has not, to our knowledge, been applied to epilepsy. PSEM^89S^ is not completely selective, as it also binds to other human receptors [17]. Indeed PSEM^89S^ has been shown to exert effects on brain function in the absence of PSAM^L141F,Y115Y^-GlyR and this was not fully reversible in vitro [62]. PSEM^89S^ is not approved for use in humans, which limits its application for imminent clinical translation [63]. Subsequent refinement of the strategy resulted in another compound, PSEM^308^, which has a much higher affinity for PSAM, with doses as low as 5 mg/kg effective in mice [64]. It has not, however, been characterised as extensively as PSAM^89S^. Magnus et al. recently further refined this system, mutating three residues in nAchRα7 and resulting in a novel PSAM (α7^L131G,Q139L,Y217F^-GlyR or PSAM4) activated by varenicline or analogues [18]. Varenicline is approved for smoking cessation by the FDA and EMA, and could be used as a repurposed ligand. It has side effects including nausea, abnormal dreams and insomnia [65,66], but it remains to be determined if it could be used for the treatment of refractory epilepsy. In addition, the reported EC_50_ of varenicline on PSAM4 (1·6 nM) is in the range of its reported effects on nAChR (α4β2 nAChR: Ki 0·4 nM; IC_50_: 2·8 nM; IC_50_ desensitization: 0·07 nM) [67,68]. The side effect profile of varenicline therefore would need to be considered before attempting chemogenetic treatment [65]. Several derivatives of varenicline have been developed as ultrapotent PSEMs (uPSEM) with very high affinity to PSAM4, but are not approved for clinical use and therefore are not suitable for imminent clinical translation.

Studies investigating the antiepileptic ability of the PSAM4/varenicline have not been reported, but the combination could represent an important step towards clinical translation, as, in common with olanzapine, the drug itself does not need to undergo extensive safety studies before licensing by the FDA/EMA. There are, however, two potential disadvantages of using a ligand-gated ion channel to treat epilepsy, in comparison with a GPCR: the principle of receptor reserve does not apply, and the chloride gradient that allows the receptor to hyperpolarize neurons can collapse. Nevertheless, the second concern should also apply to eGluCl, and yet treatment with this gene therapy was highly effective in rodent studies. Other potential obstacles to the clinical translation of PSAM4 are the need to evaluate the risk of immunogenicity to the chimeric protein, and the potential interactions of multimeric LGIC-DRs with native receptors.

## 4 Challenges and directions

Chemogenetics shows great promise not only for potential clinical application but also as a preclinical research tool to map out the central nodes of the distributed seizure networks that are increasingly being recognised in so called “focal” epilepsy. Orthogonal chemogenetic techniques could be combined to perform head-to-head comparisons of efficacy and establish the minimum brain volume required to be transduced that significantly reduces or even abolishes seizures with negligible inhibition of vital brain structures. Moreover, further preclinical studies are needed to investigate the optimum dose of activators required to sufficiently activate DRs without clinically relevant desensitization on repeated activation.

Nevertheless, clinical translation will need to overcome several potential obstacles. As well as the risks of modern gene therapy viral vectors, each specific chemogenetic DR needs to be assessed for long term efficacy and safety. Apart from the immunogenicity of non-mammalian proteins, potential heteromerization of DRs and native channel subunits should be investigated. The safety and side effects of the activator drug also need to be investigated, especially if it is not currently approved for use in humans. Finally, it should be established if the chemogenetic receptor can be activated continuously, ideally permanently, to reduce the risk of seizures irrespective of the size and location of the targeted epileptogenic zone, or if a small therapeutic window sometimes dictates that the ligand can only be used intermittently.

## 5 Outstanding questions

Despite setbacks in early clinical trials there have been several successes in recent years, leading to the first approved gene therapies such as Glybera [69], a one-time treatment for lipoprotein lipase deficiency intended to last at least ten years, and Luxturna [70], a treatment for biallelic RPE65 gene mutations that restored functional vision in clinical trials. Both of these are delivered by adeno-associated viral (AAV) vectors. A further 13 AAV-delivered treatments are currently in phase 3 clinical trials, including LYS-SAF302, a treatment for Mucopolysaccharidosis Type IIIA given via intracerebral injections [71]. As viral vector technology has matured it is increasingly evident that gene therapy can be safe, but questions remain about the maintenance of efficacy many years after treatment. Limited data in primates have shown persistent expression fifteen years following intracerebral AAV injection [72], and a recent phase 1 clinical trial of patients with Parkinson's disease reported functional improvement lasting at least twelve months [73]. Now that several candidate chemogenetic treatments have emerged, the prospect of a single-shot cure for resistant epilepsy is getting ever closer [74]. The time is now ripe for a clinical trial of gene therapy in epilepsy, and chemogenetics shows considerable promise because of the ability to tune the therapeutic effect.

## 6 Search strategy and selection criteria

Data for this Review were identified by searches of MEDLINE, Current Contents and PubMed and using relevant keywords, and additional articles as found in the initial search. Recent abstracts were also included due to the highly relevant nature of their claims. Only articles published in English between 1980 and 2019 were included.

## Funding

This work was supported by Brain Research UK, European Union's Horizon 2020 research and innovation program (Marie Skłodowska-Curie grant agreement no. 701411 to A.L.), the Wellcome Trust, and the Medical Research Council. The funders had no role in paper design, data collection, data analysis, interpretation or writing of the paper. No funding was received from any pharmaceutical company or other agency to write this paper.

## Conflict of interest

Dr. Lieb reports grants from European Union's Horizon 2020 research and innovation program (Marie Skłodowska-Curie grant agreement no. 701411 to A.L.), grants from Wellcome Trust, grants from Medical Research Council, during the conduct of the study; .

Dr. Weston reports grants from Brain Research UK, during the conduct of the study; .

Dr. Kullmann reports grants from Wellcome Trust, grants from Medical Research Council, during the conduct of the study; In addition, Dr. Kullmann has a patent “Combined use of a vector encoding a modified receptor and its exogenous agonist in the treatment of seizures” pending.

## Author contributions

A.L., M.W. and D.M.K all contributed equally to conceptualising and writing the original manuscript, creating the figures and writing the revisions of this review.
